# An image encryption scheme using 4-D chaotic system and cellular automaton

**DOI:** 10.1038/s41598-025-95511-y

**Published:** 2025-06-04

**Authors:** Ibrahim Al-Dayel, Muhammad Faisal Nadeem, Meraj Ali Khan, Bahreselam Sielu Abraha

**Affiliations:** 1 Department of Mathematics and Statistics, College of Science, Imam Mohammad Ibn Saud Islamic University (IMSIU), Riyadh, 11566 Saudi Arabia; 2 Department of Mathematics, COMSATS University Islamabad, Lahore Campus, Lahore, 54000 Pakistan; 3 Mainefhi College of Engineering and Technology, Mainefhi, Asmara Eritrea

**Keywords:** Image encryption, Chaos-based cryptography, Chaotic keystream generation, Cellular automaton, Security analysis, Four-dimensional chaotic system, Mathematics and computing, Computer science

## Abstract

This paper proposes an innovative image encryption scheme exploiting the chaotic nature of a four-dimensional chaotic system and the computational capability of Langton’s Ant cellular automaton. Traditional three-dimensional chaotic systems often have restricted key space and limited complexity, making them vulnerable to cryptanalysis. To address these limitations, the proposed scheme integrates multi-layered transformations, including chaotic diffusion, symbolic encoding, and dynamic keystream generation. Comprehensive security analyses demonstrate that the proposed scheme achieves near-optimal results, including a large key space of approximately 1$10^{840}$, high entropy (7.9977), and excellent differential attack resistance indicated by NPCR ( 99.61%) and UACI ( 33.44%) metrics. The proposed method effectively disrupts pixel correlations, providing robust protection against various cryptographic threats. These results confirm that our encryption approach offers a secure, efficient, and practical solution for protecting multimedia data in modern digital communication systems.

## Introduction

Cryptography is one of the cornerstones behind modern digital security, covering confidentiality, integrity, and authenticity of data in today’s increasingly connected world^1,2^. Be it safeguarding online financial transactions^3^ or enabling private communications, cryptographic systems find indispensable applications in both civilian and military worlds^4^.

The exponential growth in digital communication technologies has made the transmission of multimedia data an indispensable part of modern life. While such growth in digital communication technologies has raised the need for robust mechanisms of data security, especially in image encryption, the uniqueness of images as compared to text is their high redundancy, bulk size, and strong correlation between adjacent pixels. These features make it impossible for standard cryptographic algorithms^5,6^, which usually are developed to fit text data, to efficiently meet the challenges laid down by them. This makes the development of image data-specific encryption algorithms an important current priority in information security research.

Chaotic systems have come out to be promising ground for developing secure cryptographic algorithms^7^. Chaotic systems possess all the properties required for getting secure encryption, such as sensitivity to initial conditions, randomness that is deterministic, and unpredictability for long terms. Various works have taken advantage of the capabilities of low-dimensional chaotic maps for generating pseudo-random sequences; these include logistic maps, tent maps, and sine maps. But usually, those low-dimensional mapping functions suffer from some drawbacks on the limited key space and less complexity, so that it can be vulnerable under various kinds of attack. The use of higher-order chaotic systems considers providing higher-order complexity with large key space and thus security^8^.

Another fascinating attack, much less intuitively computational in nature, is a variety of a simple but powerful cellular automaton and has been to do with Langton’s Ant - a variant of a two-dimensional Turing machine. Toughi et al. proposed an image encryption scheme that combined elliptic curve pseudo-random sequences with AES to enhance security and computational efficiency^9^. In the respective grid, it acts to modify its state according to the direction changes dictated by the deterministic instructions followed by the Langton ant. Being so naive, after several moves, it weaves a fairly complicated pattern thus objectifying computational one-way processes. This property makes it the right candidate for scrambling images and confusing processes. Also, when combined with chaotic systems, Langton’s Ant can further extend this cryptographic strength by adding even more layers of unpredictability and hence complexity.

Nowadays, image encryption finds a wide range of applications in various fields of interest, including secure communications, medical imaging, and military operations. In recent years, a number of techniques have been developed to meet the demand for secure, efficient, and robust methods of encryption. Chaos-based systems, parallel processing, and innovative key management strategies have significantly enhanced this field. This increases the requirement for strong image encryption techniques in order to maintain privacy, with an ever-growing dependency on digital communication and multi-media applications. Encryption transforms the plain images into some coded forms that enable only the authorized to decrypt them. There are, however, problems in managing the big size of the image data and special features, such as pixel correlations and redundancies, theoretically applicable in classical text-based traditional methods. The main propelling factors to develop the particular encryption technique in respect of an image are that different methodologies will find difficulties in providing all-round efficient performances in different types.

In order to reduce computational complexity, the Chaotic Dynamical State Variables Selection Procedure developed by Bashir et al. is adopted to select Chaotic Dynamical State Variables Selection Procedure (CDSVSP). More precisely, for encrypting a color image of size, more than chaotic variables in each encryption process should be used for maintaining high security^10^. Malik et al. presented a color image encryption algorithm based on the hyperchaotic systems and DNA computing for strong encryption^11^.

Man et al. proposed a double-image encryption algorithm based on convolutional neural networks and dynamic adaptive diffusion^12^. Chaos maps are used in the initial conditions, while CNN-generated chaotic pointers for scrambling have been utilized in the proposed scheme, which enhances its robustness against various plaintext attacks. Mirzaei et al. proposed a parallel sub-image encryption method that decomposes the plain image into four sub-images. To address computational challenges, researchers have focused on parallel processing and low-complexity designs^13^. Chen et al. presented a fast chaos-based image encryption scheme using a dynamic state variables selection mechanism for enhanced performance^14^. Wang et al. presented a parallel image encryption algorithm which used a cyclic shift and sorting together for permutation along with a new kind of parallel diffusion method^15^. Zhou et al. proposed a novel bitplane-based encryption algorithm in which key bitplanes could be selected by the users for encryption^16^. Fu et al. had proposed a chaos-based digital image encryption scheme based on an enhanced diffusion strategy, which provides greater security levels^17^. However, it cannot be said to be secure because frequency analysis attacks were not considered.

Zhang and Xiao presented a rotation grid and block distribution method for increasing the randomness of pixels in encrypted images^18^. Rhouma et al. proposed a piecewise linear chaotic map for improving robustness in image encryption by transforming vectors uniquely^19^. Van Droogenbroeck and Benedett proposed selective encryption for the significant bit planes that can ensure efficiency with acceptable visual corruption^20^. Wang et al. proposed the chaos-based image encryption algorithm with variable control parameters, which can enhance encryption adaptability and robustness^21^. Sinha and Singh proposed the 3-D jigsaw transformation with a fractional Fourier transformation for robust encryption in frequency-domain^22^. Ramakrishna et al. presented an elliptic curve cryptography-based secure and authenticated image encryption scheme that assures confidentiality and authentication^23^. El-Latif et al. worked on the passive approach of the detection of image splicing based on the deep learning-wavelet transforms^24^. Ye et al. proposed a new double image encryption framework that contains compressive sensing and elliptic curve cryptography to offer both higher data reduction and security^25^. Abuturab presented a color information security system based on DCT in the gyrator transform domain with radial-Hilbert phase encoding^26^. Hu et al. proposed a chaotic image cryptosystem using DNA deletion and insertion operations to enhance the complexity of encryption^27^. Taneja et al. integrated spatial wavelets and Arnold Cat transformations for partial encryption of critical image components^28^. Norouzi and Mirzakuchaki came up with the image encryption algorithm based on DNA sequence operations along with a cellular neural network, whereby the security for encryption was improved accordingly^29^. Patidar et al. proposed a robust and secure image encryption scheme based on chaotic standard maps,^30^.

Recent advances in image encryption prove a significant trend toward chaos-based techniques. Liu et al. introduced various schemes that exploit chaotic maps, dynamic S-box constructions, and cipher feedback mechanisms to enhance security in color image encryption^31–34^. Feng et al. further extended these ideas by proposing multi-image encryption methods and secure transmission schemes leveraging hyperchaotic systems and fractional-order dynamics^35–38^. In parallel, Alexan and co-authors integrated unconventional techniques such as DNA coding and cellular automata, expanding the design space for secure image cryptosystems^39–43^.

Complementing these encryption schemes, research on noncommutative cryptosystems has broadened the theoretical foundation of secure communications^44–46^. Moreover, critical cryptanalysis of chaos-based encryption schemes has identified vulnerabilities and motivated the development of more robust methods^47,48^. Recent works continue to refine these approaches by addressing emerging challenges in both image encryption and secure key exchange^49–53^.

Yang and Kim proposed a 3D phase encoding scheme for the security of the biometric images by using holographic interference patterns^54^. Bashir et al. proposed a 4D chaos-based encryption mechanism that used dynamic state variable selection for pixel-level confusion and diffusion^55^. Mastan et al. proposed hybrid encryption using discrete wavelet transforms and chaotic systems for secure storage of multimedia^56^. A new layer-based image encryption framework was put forward by Rad et al., which incorporates multiple efficient encryption algorithms and provides variable security for image blocks concerning their significance to balance the computational cost-security ratio^57^. Experimental analyses demonstrated improved metrics of entropy and correlation with no increase in computational time. Su et al. proposed a model dealing with 3D space permutation and diffusion and incorporating DNA coding for improved sensitivity in encryption^58^.

In this paper, we introduce the new image encryption scheme using a four-dimensional chaotic system and Langton’s Ant cellular automaton. The proposed encryption technique ensures security on multiple layers through both the transformation and permutation/diffusion processes. Our algorithm achieves a ciphertext with very high entropy and eliminates pixel correlations, making the proposed algorithm resistance against various kinds of cryptographic attacks. Several intensive simulations were performed to confirm that our method provides high diffusion, sensitivity to the initial conditions, statistical, and differential attack resistance. The mathematical basis, algorithm design, implementation, and full security analysis are given in the following sections, followed by the discussion of experimental results and comparative evaluation. Our choice of a four-dimensional (4D) chaotic system in our encryption algorithm is motivated by its intrinsic advantages over traditional three-dimensional (3D) chaotic systems such as Lorenz or Chen systems. Specifically, 4D chaotic systems provide much wider key spaces, which lead to a high resistance to brute-force attacks. Moreover, with more dimensions comes hyperchaotic behavior, with a higher complexity and richer dynamics and more initial-condition sensitivities, making the algorithm more secure against cryptanalytic attacks. This added complexity also ensures improved diffusion and confusion properties, which are essential in efficiently securing image data against statistical and differential attacks. The remainder of this paper is organized as follows: Section “Preliminaries” provides essential preliminaries, detailing the foundational concepts of the four-dimensional chaotic system, the chaotic tent map, and the cellular automaton dynamics employed. Section “Proposed encryption scheme” describes our proposed encryption methodology, including preprocessing steps, keystream generation, and the multi-stage encryption process. Section “Simulation” discusses the theoretical merits of our proposed method, emphasizing its complexity and cryptographic advantages. In “Discussion”, extensive simulation results and experimental validations are presented, while “Security analysis“ offers comprehensive security analyses addressing resistance to brute-force, statistical, and cryptanalytic attacks. Finally, “Conclusion” concludes the paper, summarizing key findings and proposing directions for future research.

The images used in the paper are available in the USC-SIPI Image Database, which can be accessed online at http://sipi.usc.edu/databas (Weber, A. G. (2006). The USC-SIPI image database: Version 5. http://sipi.usc.edu/database/).

## Preliminaries

The proposed image encryption scheme will be based on some foundational concepts and techniques reviewed in this section, presenting the four-dimensional chaotic system, Langton’s Ant cellular automaton, and cryptographic operations of permutation, confusion, and diffusion. In fact, they are the core of the encryption algorithm that ensures security due to its inherent randomness and complexity.

### Four-dimensional chaotic system

Chaotic systems are mathematical models that have deterministic behaviors, but with sensitive dependence on initial conditions. The intrinsic unpredictability and ergodic properties of chaotic systems make them very suitable for cryptographic applications, especially in pseudo-random sequence generation and key management. In this paper, we will use a four-dimensional chaotic system introduced by Yong and Yun-Qing^59^, described by the following equations

1 $$\begin{aligned} \left\{ \begin{aligned} y_1'&= p \cdot y_1 - c_1 \cdot y_2 \cdot y_3 \cdot y_4, \\ y_2'&= q \cdot y_2 - c_2 \cdot y_1 \cdot y_3 \cdot y_4, \\ y_3'&= r \cdot y_3 - c_3 \cdot y_1 \cdot y_2 \cdot y_4, \\ y_4'&= s \cdot y_4 - c_4 \cdot y_1 \cdot y_2 \cdot y_3. \end{aligned}\right. \end{aligned}$$

**Figure 1:**
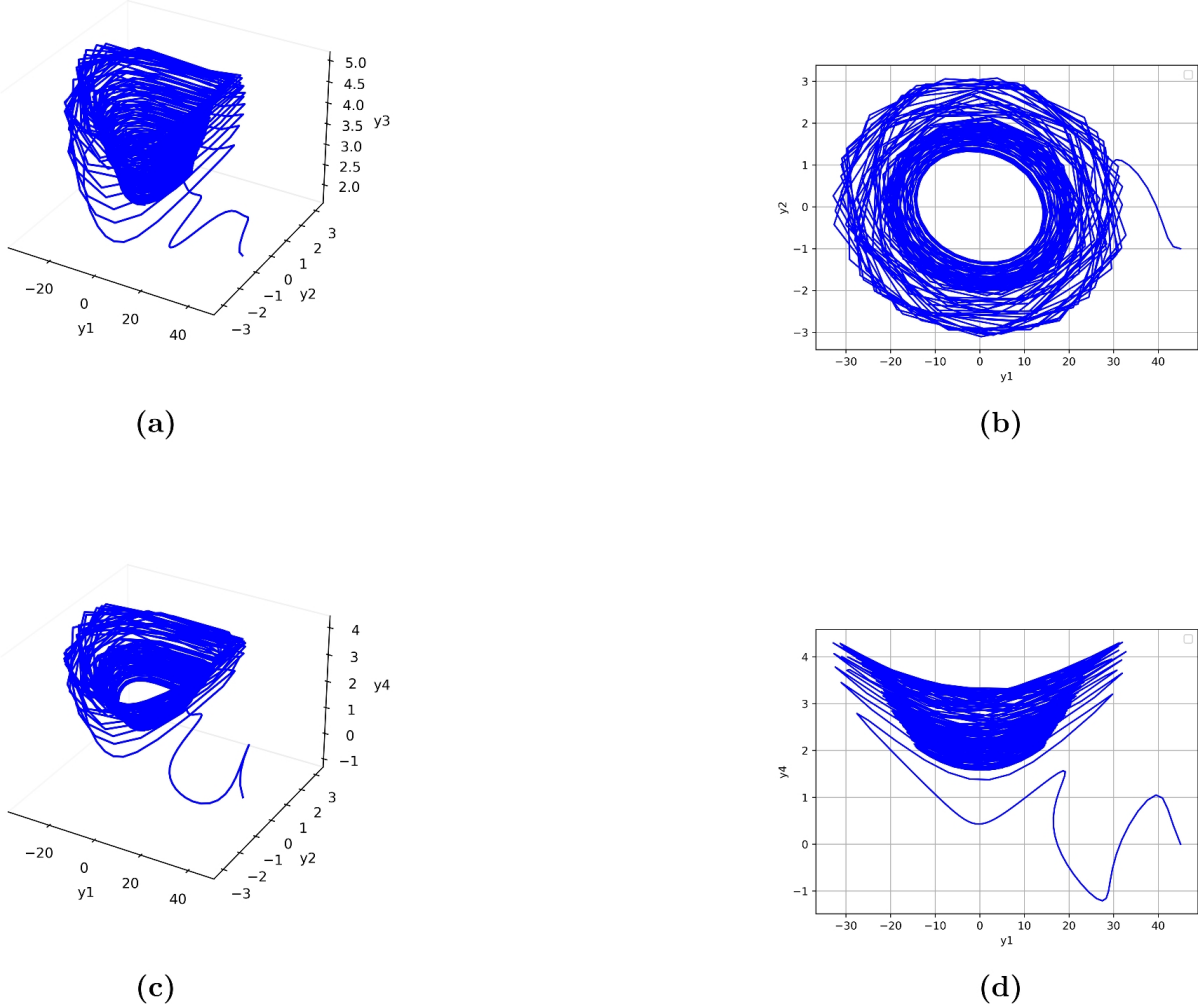
Chaotic behavior of dynamical system.

Here, $y_1, y_2, y_3, y_4$ are state variables, and $p, q, r, s, c_1, c_2, c_3, c_4$ are system parameters that govern the system’s dynamics. These equations, implemented in MATLAB, generate a high-dimensional chaotic sequence with increased complexity compared to traditional low-dimensional systems. Figure 1 shows the chaotic behavior of the attractors the four dimensional chaotic dynamical system.

This sequence is used for generating keys, pixel permutation, and driving the dynamic rules of Langton’s Ant.

### Chaotic tent map

The tent map is a piecewise-defined chaotic function that has been used to generate random data in several chaos-based applications, including image encryption schemes. The form of the tent map used is defined as^60^

2 $$\begin{aligned} f(u, \eta , v) = {\left\{ \begin{array}{ll} \big \lceil \frac{\eta }{u} v\big \rceil , & \text {if } 0 \le v \le u, \\ \left\lfloor \frac{\eta (\eta - v)}{\eta - u}\right\rfloor + 1, & \text {if } u < v \le \eta , \end{array}\right. } \end{aligned}$$

where $\eta$ and $u$ are parameters controlling the map’s behavior. $v$ is the current state variable, constrained within the range $[0, \eta ]$. $u \in (0, \eta )$, and it is an integer.

### Chaotic dynamical state variables selection procedure

The most relevant factors in ensuring cryptographic strength of the proposed encryption scheme are the selection and utilisation of chaotic state variables. The sequences generated by the chaotic system, described by four-dimensional differential equations, have all properties of high sensitivity for initial conditions, deterministic randomness, and long-term unpredictability. Following, this section provides in detail the methodology adopted to choose the state variables of chaos efficiently, inspired by the CDSVSP presented in related works.

#### Selection procedure

To efficiently utilize chaotic data and reduce computational overhead, we adapt the CDSVSP procedure as follows

**Generate Chaotic Data:** The chaotic system is iterated for $n_0 + \frac{MN}{4}$ steps (with $n_0 \ge 100$) to ensure the initial transients are eliminated. Here, $M \times N$ is the total number of pixels in the input image.

**Define the State Set:** At each iteration, the system produces four state variables, $\{X, Y, Z, W\}$. A one-dimensional array of pixel values $P = \{P(0), P(1), \dots , P(MN-1)\}$ is used to map these states.

**Selection Mechanism:** For each pixel $P(i)$, a dynamic indicator determines the selected variable $S(i)$ from the state set. The indicator, $index~(i)$, is calculated as

$$\begin{aligned} index~(i) = \text {mod}(\text {tent}(u, \eta , P(i-1)), 4), \end{aligned}$$

where the tent map introduces randomness, and $u, \eta$ are secret parameters contributing to the key.

**State Update:** Based on $index~(i)$, the chaotic system’s state set is updated as

3 $$\begin{aligned} X(i+1) = {\left\{ \begin{array}{ll} \{X_{i+1}, Y_i, Z_i, W_i\}, & \text {if } index~(i) = 0, \\ \{X_i, Y_{i+1}, Z_i, W_i\}, & \text {if } index~(i) = 1, \\ \{X_i, Y_i, Z_{i+1}, W_i\}, & \text {if } index~(i) = 2, \\ \{X_i, Y_i, Z_i, W_{i+1}\}, & \text {if } index~(i) = 3. \end{array}\right. } \end{aligned}$$

### Langton’s ant cellular automaton

In addition to the ongoing chaotic dynamics provided by the four-dimensional system, Langton’s Ant is incorporated into the encryption scheme to add another layer of symbolic transformation. While the overall purpose of the chaotic system is to generate complex, high-dimensional sequences, Langton’s Ant operates on the basis of a set of certain rules that, when iteratively applied, lead to highly non-linear and unpredictable symbolic patterns. This discrete, rule-based process enhances the confusion and diffusion properties of the scheme. In effect, the incorporation of Langton’s Ant complements the continuous chaos by making even minor changes to the input lead to highly different symbolic outputs. Rather than simply adding to the chaotic nature, the inclusion of Langton’s Ant greatly improves the overall security of the encryption process by combining the benefits of continuous and discrete chaotic systems. Langton’s Ant has the reputation of being among the most chaotic and dynamic cellular automaton. Because its movements are determined though well-nigh unpredictable, this automaton will create new ways of inserting confusion and diffusion into the scheme for encryption. The rule-based mapping of pixel values, which is dynamic in nature, thus helps to incorporate additional security and robustness within the algorithm through the use of Langton’s Ant.

Langton’s Ant is a two-dimensional Turing machine that moves on a grid according to simple rules. It is defined by

**States:** Each cell in the grid is either black or white.

**Rules about Movement:**

1. If the ant stands on a white cell, which changes color to black before moving forward by one step $90^{\circ }$ to the right.
2. If the ant is on a black cell, it rotates 90$^{\circ }$ left, then changes the color of that cell to white and moves.

#### Langton’s ant rule

The conceptual framework of the Langton’s Ant represents this process of encryption in which the pixel data gets encoded and manipulated symbolically. To be specific, mapping pixel values into sequences of ‘L’ and ‘R’ characters relies on rules dependent on chaotic systems and key parameters.

**Mapping Decimal to Ant Rules:** Each pixel value - an 8-bit decimal number between 0 and 255 is first converted to its binary representation and then mapped to sequences of ‘L’ and ‘R’. This mapping depends on a rule number, which is derived from a chaotic keystream or another key-dependent mechanism. Rule number generation, For each pixel index  $i$, the rule number is computed as

$$\begin{aligned} r(i) = \text {mod}(l_1(i), 8) + 1 \end{aligned}$$

where $l_1(i)$ is a key stream value generated using the chaotic data.

**Figure 2:**
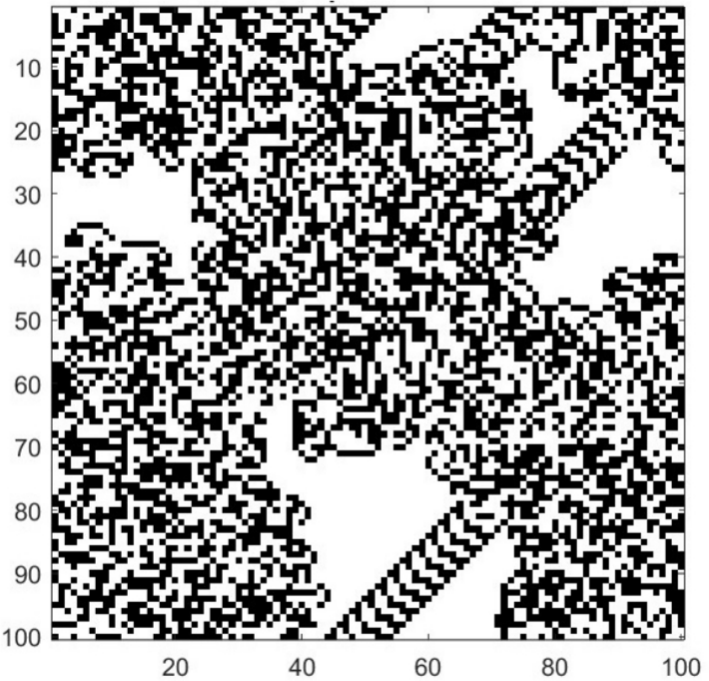
Langton’s ant after 100,000 steps.

Figure 2 shows the Langton’s ant after 100,000 steps. After some time, the Langton’s Ant starts to develop chaotic behavior in the form of unpredictable paths and complex patterns.

** Binary to Ant Rule Mapping** For each rule $r(i)$, the mapping of binary pairs to ‘L’/‘R’ is defined in Table 1.

**Table 1:** Binary to ant rule mapping.

Binary pair	Rule 1	Rule 2	Rule 3	Rule 4	Rule 5	Rule 6	Rule 7	Rule 8
00	L	R	L	R	L	R	L	R
01	R	L	L	R	R	L	L	R
10	L	R	R	L	R	L	L	R
11	R	L	R	L	L	R	R	L

For each pixel, convert the decimal pixel value into an 8-bit binary string. The binary string is divided into four 2-bit pairs. Each pair is mapped to ‘L’ or ‘R’ based on the rule number chosen. For example, for 156 as the pixel value and for rule number 3, binary ‘10011100’, pairs ‘10, 01, 11, 00.’ Mapping as per Rule 3: $10 \rightarrow R, 01 \rightarrow L, 11 \rightarrow R, 00 \rightarrow L.$ The resultant ant rule sequence is *RLRL*.

After the decimal-to-ant conversion, antrule sequences are diffused by symbolic operations. Three major operations are defined. Ant XOR applies a symbolic XOR operation on two ant-rule sequences $A$ and $B$. Each corresponding character in $A$ and $B$ is combined as

$$\begin{aligned} L \oplus L = L, \quad L \oplus R = R, \quad R \oplus L = R, \quad R \oplus R = L. \end{aligned}$$

Ant addition combines two sequences bitwise modulo 2,

$$\begin{aligned} L + L = L, \quad L + R = R, \quad R + L = R, \quad R + R = L \end{aligned}$$

Ant subtraction performs the usual subtraction modulo 2,

$$\begin{aligned} L - L = L, \quad L - R = R, \quad R - L = R, \quad R - R = L \end{aligned}$$

This all ensures that ant-rule sequences do not become non-linearly mixed across channels.

## Proposed encryption scheme

In this section, we present the detailed methodology of the Proposed Image Encryption Scheme (PIES), which effectively integrates a four-dimensional chaotic system and a cellular automaton to ensure high security. To enhance readability and clarity, we have structured the encryption process into distinct, clearly defined steps, preprocessing and initial chaotic data generation, chaotic keystream generation, pixel permutation, symbolic-level encoding using cellular automaton rules, multi-stage diffusion (both decimal and symbolic), and reconstruction of the encrypted cipher image. Utilizing these multi-level transformations-decimal-level diffusion, chaotic permutation, and symbolic representation using cellular automata-our scheme of encryption significantly enhances the resilience to security, with outstanding resistance to statistical, differential, and brute-force attacks. A simple flowchart have also been provided to describe these steps of the encryption to make it more readable and reproducible.

### Preprocessing and chaotic data generation

Given a $M \times N$ colour image with Red (R), Green (G) and Blue (B) channels, each channel converts to a one-dimensional vector of length $MN$. Denote these vectors by

4 $$\begin{aligned} \left\{ \begin{aligned} RC&= \{RC(0), RC(1), \ldots , RC(MN-1)\},\\ GC&= \{GC(0), GC(1), \ldots , GC(MN-1)\},\\ BC&= \{BC(0), BC(1), \ldots , BC(MN-1)\} \end{aligned} \right. \end{aligned}$$

Initialize a 4D chaotic system with secret keys $(x_0, y_0, z_0, w_0)$. Numerically integrate this system for $(MN)/4 + n_0$ iterations, where $n_0 \ge 200$. This produces a set of chaotic values

$$\begin{aligned} D = \{D_1, D_2, D_3, D_4\} \end{aligned}$$

where each $D_i$ is a vector of chaotic values for each dimension in the system, these will serve as sources of randomness for subsequent processes.

### Key stream generation procedure

The proposed image encryption algorithm will generate secure and random key streams using a Key Stream Generation Procedure (KSGP) based on the chaotic system and Langton’s Ant dynamics. KSGP will ensure that very small changes in initial conditions or input data generate completely different key streams, hence enhancing cryptographic strength. The keystream generation utilizes the chaotic sequences generated by a four-dimensional chaotic system with secret parameters $x_0, y_0, z_0, w_0$. The red, green, and blue pixel channels of the input image ($RC, GC, BC$) are first unraveled into one-dimensional arrays of size $MN$. These are further combined into a single array $S$ using a linear combination with secret integer coefficients $\sigma _1, \sigma _2, \sigma _3$ as

$$\begin{aligned} S = \text {mod}(\sigma _1 \cdot RC + \sigma _2\cdot GC +\sigma _3 \cdot BC, 256). \end{aligned}$$

That gives, for every index of a pixel, the following key-dependent chaotic map is used in order to calculate the selection state *ss*

$$\begin{aligned} ss = \text {mod}(\text {tent}(key1, key2, S[i]), 4). \end{aligned}$$

Nonlinear transformations are dictated by the tent map. According to $ss$, each of the four chaotic series chose the respective counter $n_1, n_2, n_3, n_4$. Then, the chaotic value $st(i)$ is selected from the selected sequence using

$$\begin{aligned}st(i)=D_{ss+1}[200 + n_{ss+1}].\end{aligned}$$

**Figure 3:**
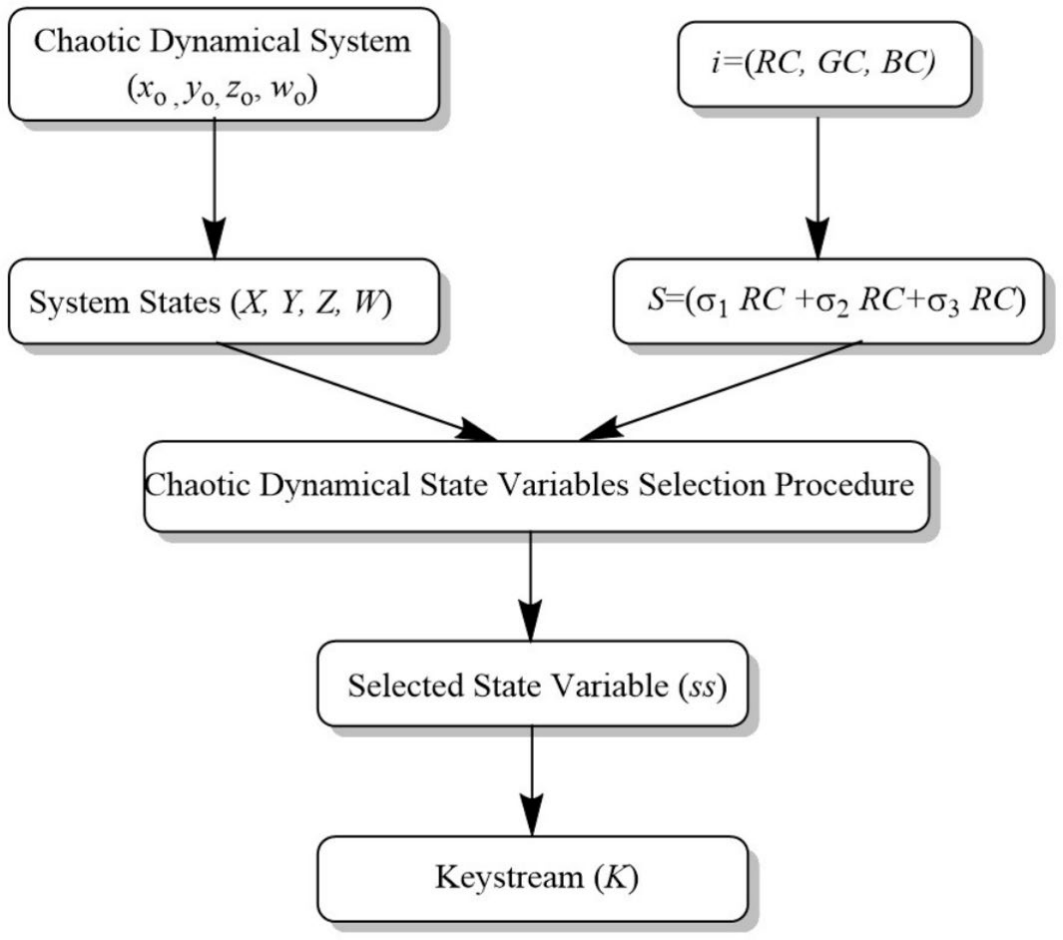
Keystream generation procedure.

Then, the fractional part of this chaotic value is extracted, multiplied by $10^{15}$, and then reduced modulo 256 using

$$\begin{aligned}K(i) = \text {mod}(\lfloor (|st(i)| - \lfloor |st(i)| \rfloor ) \times 10^{15} \rfloor , 256)\end{aligned}$$

to generate an 8-bit keystream value. The above-mentioned process is iteratively done for each MN pixel in order to prepare a keystream array $K={K(0),K(1),\dots ,K(MN-1)}$ to perform pixel-level encryption in a totally secure manner. High key sensitivity and sensitivity on the initial conditions ensures resistance from any kind of cryptanalysis attack. Figure 3 shown the key stream generation procedure.

### Encryption steps

This encryption scheme follows a series of transformations that will result in robust security due to the combination of chaotic keys, dynamic rule-based mapping, and operations from Langton’s Ant. The transformation, therefore, assures heavy confusion and diffusion of pixel values. The detailed steps for encryption are given as follows.

**Step 1: Initial Decimal Diffusion**

First step - mix of pixel values from input channels ($RC, GC, BC$) with chaotic keystreams and constants for good initial diffusion. For each pixel at index  $i$,

1. For $$i = 0$$

5 $$\begin{aligned} \left\{ \begin{aligned} RC_{1}(i)&= (RC(i) + K_{1}(i) + \sigma _{1} + K_{2}(i)) \mod 256,\\ GC_{1}(i)&= (GC(i) + K_{2}(i) + \sigma _{2} + RC_{1}(i)) \mod 256,\\ BC_{1}(i)&= (BC(i) + K_{3}(i) + \sigma _{3} + GC_{1}(i)) \mod 256. \end{aligned} \right. \end{aligned}$$

2. For $$i > 0$$

6 $$\begin{aligned} \left\{ \begin{aligned} RC_{1}(i)&= (RC(i) + K_{1}(i) + RC_{1}(i-1) + K_{2}(i)) \mod 256,\\ GC_{1}(i)&= (GC(i) + K_{2}(i) + GC_{1}(i-1) + RC_{1}(i)) \mod 256,\\ BC_{1}(i)&= (BC(i) + K_{3}(i) + BC_{1}(i-1) + GC_{1}(i)) \mod 256. \end{aligned} \right. \end{aligned}$$

This process introduces chained dependencies between pixel values and chaotic keys, amplifying the diffusion effect.

Output: Arrays $$RC_{1}, GC_{1}, BC_{1}$$.

**Step 2: Permutation of Pixels**

To disrupt the spatial correlations in the image, permutation is applied

1. Combine $RC_{1}, GC_{1}, BC_{1}$ into a single array $T$ of size $3MN$

$$\begin{aligned} T = \{RC_{1}(0), \dots , RC_{1}(MN-1), GC_{1}(0), \dots , GC_{1}(MN-1), BC_{1}(0), \dots , BC_{1}(MN-1)\}. \end{aligned}$$

2. Use a chaotic tent map to compute a permutation vector $\sigma$ based on chaotic keys. Reorder the indices of $T$ according to $\sigma$

$$\begin{aligned} T'(\sigma (i)) = T(i). \end{aligned}$$

3. Split $T'$ back into three separate arrays

$$\begin{aligned} RC_{2} = T'(0:MN-1), \quad GC_{2} = T'(MN:2MN-1), \quad BC_{2} = T'(2MN:3MN-1). \end{aligned}$$

Output: Arrays $$RC_{2}, GC_{2}, BC_{2}$$.

**Step 3: Symbolic (Ant-Rule) Encoding and Diffusion**

1. Decimal-to-Symbolic Conversion: Each pixel in $RC_{2}, GC_{2}, BC_{2}$ is mapped to Langton’s Ant movement sequences ($`L'$ for left, $`R'$ for right). The mapping is determined by chaotic keys, which select a dynamic rule for encoding

$$\begin{aligned} RC_{2}(i) \xrightarrow [\text {rule-dependent}]{\text {convert}} RC_{3}(i) \in \{\text {L,R sequences}\}. \end{aligned}$$

Similar conversions are applied to $GC_{2}$ and $BC_{2}$.

2. Ant-Rule Level Diffusion: The symbolic arrays $RC_{3}, GC_{3}, BC_{3}$ are diffused by applying symbolic operations. These operations are analogous to binary XOR, addition, and subtraction, but they act on sequences of $`L'$ and $`'$. For each pixel: - Select an operation type based on chaotic keys. - Combine symbolic sequences from one channel with another to generate a new, diffused sequence.

Output: Symbolic arrays $RC_{3}, GC_{3}, BC_{3}$.

**Step 4: Symbolic-to-Decimal Re-Conversion**

The symbolic sequences from the previous step are mapped back into decimal pixel values. The reconversion uses chaotic keys to determine the mapping for each sequence

$$\begin{aligned} RC_{3}(i) \xrightarrow [\text {rule-dependent}]{\text {convert}} RC_{4}(i), \end{aligned}$$

and similarly for $GC_{3}$ and $BC_{3}$.

Output: Arrays $$RC_{4}, GC_{4}, BC_{4}$$.

**Step 5: Final Decimal Diffusion**

A final diffusion step is applied to the arrays $RC_{4}, GC_{4}, BC_{4}$ to further mix the pixel values. This step is similar to the initial decimal diffusion but uses transformed pixel values

7 $$\begin{aligned} \left\{ \begin{aligned} RC_{5}(i)&= (RC_{4}(i) + K_{4}(i) + RC_{5}(i-1)) \mod 256,\\ GC_{5}(i)&= (GC_{4}(i) + K_{5}(i) + GC_{5}(i-1)) \mod 256,\\ BC_{5}(i)&= (BC_{4}(i) + K_{6}(i) + BC_{5}(i-1)) \mod 256. \end{aligned} \right. \end{aligned}$$

Output: Arrays $$RC_{5}, GC_{5}, BC_{5}$$.

**Step 6: Cipher Image Reconstruction**

Finally, the arrays $RC_{5}, GC_{5}, BC_{5}$ are reshaped into their original $M \times N$ dimensions

$$\begin{aligned} R' = \text {reshape}(RC_{5}, M, N), \quad G' = \text {reshape}(GC_{5}, M, N), \quad B' = \text {reshape}(BC_{5}, M, N). \end{aligned}$$

The channels are stacked to produce the encrypted image

$$\begin{aligned} I_{cipher} = \{R', G', B'\}. \end{aligned}$$

Output: The final encrypted image $I_{cipher}$.

**Figure 4:**
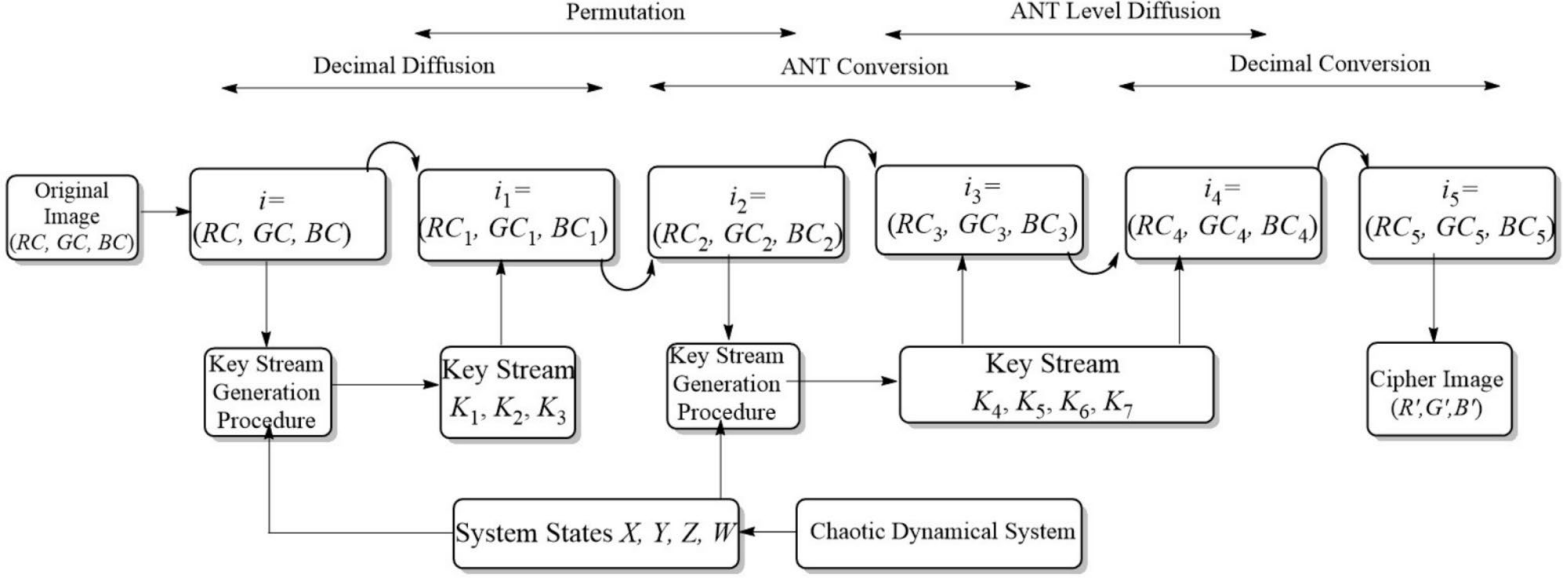
Encryption procedure.

The flow chart of encryption procedure is shown in Fig. 4.

## Simulation

The proposed encryption scheme has been coded and compiled using MATLAB R2020a running on Microsoft Windows 11 Home (64-bit, Version 10.0.26100), powered by an AMD Ryzen 9 8945HS processor with integrated Radeon 780M graphics (Firmware version M5406UA.310, released on 2024-10-24) and 16GB of RAM. In the domain of image cryptography, robust security and resistance against common attacks are very important challenges. Chosen plaintext/ciphertext attack, statistical attack, differential attack, brute force attack, and so on pose serious challenges in the design of secure encryption algorithms. Therefore, the main objective of any encryption technique is to show resilience against these types of threats while maintaining computational efficiency.

**Figure 5:**
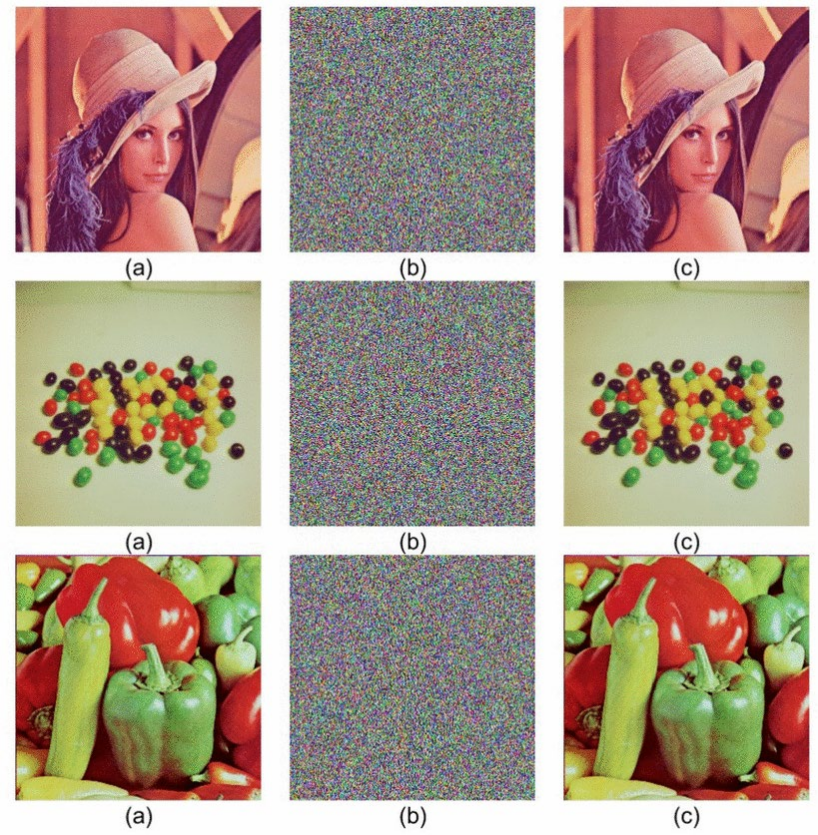
(**a**) Original (**b**) Encrypted (**c**) Decrypted images.

In order to assess the performance and security of the proposed encryption algorithm, several color images from the USC-SIPI Image Database are considered. The images used include ‘Lena,’ ‘Beans,’ and ‘Peppers,’. These are all resized to a standard resolution of $256\times 256$ pixels. All the simulations have been carried out in MATLAB R2023a using double-precision computations as per the IEEE 754 standard for numerical accuracy.

In order to simulate the proposed encryption algorithm, initial values and system parameters are selected for the four-dimensional chaotic system as $x_0 = 19, \, y_0 = 37, \, z_0 = 113, \, w_0 = 12, \, p = -7, \, q = 3, \, r = -1.2, \, s = -2, \, c_1 = 2, \, c_2 = -1, \, c_3 = 1, \, c_4 = 1.$ Great care has been taken in choosing the step size for solving the chaotic system to be small enough to provide numerical stability and avoid any unwanted chaotic degradation during simulations.

Each of the test images shown in Fig. 5a was then applied with the encryption process and correspondingly encrypted and decrypted images were obtained. Figure 5b results show that the encryption process completely destroyed the visual structure of the original images for the sake of confidentiality. The decrypted images shown in Fig. 5c are perfectly reconstructed with the use of correct secret keys, which validates the reversibility and accuracy of the algorithm. Any slight modification in the initial keys leads to a failure in decryption, showing that the proposed method is highly sensitive to the key.

## Discussion

The proposed image encryption algorithm provides a fresh and robust approach toward image security with a four-dimensional chaotic system in conjunction with Langton’s Ant cellular automaton. In fact, inherent sensitivity of initial conditions and unpredictability are exclusive characteristics of chaos that may combine with the complex and, at the same time, deterministic behavior of Langton’s Ant to enhance processes for confusion and diffusion towards attaining high-level security that is resistant to any sort of cryptanalytic attack.

The use of a four-dimensional chaotic system significantly increases the algorithm’s complexity, compared to traditional low-dimensional chaotic models. This provides a large key space and high sensitivity to initial conditions, making it highly resistant against brute-force attacks. Chaotic systems drive the permutation and diffusion by pseudo-random sequences, hence the generated outputs are completely different even for small changes in the initial parameters. This sensitivity is one of the most important features for preventing chosen-plaintext and known-plaintext attacks.

This ant cellular automaton improves symbolic-level operation in the whole process of encryption. In image encryption, the pixel value is changed into ant sequences of rules ‘L’ and ‘R’ via algorithms. It essentially just adds another layer of difficulty - obfuscation. These ensure strong randomness and uniformity of the encrypted image, symbolic diffusions such as Ant XOR, addition, and subtraction. These symbolic operations disrupt the statistical pattern to avoid statistical and differential attacks.

It provides strong security without sacrificing computational efficiency. Fast key generation and dynamic pixel mapping can be achieved based on the mathematical properties of the chaotic system. The tent map provides randomness for pixel permutation and the selection of rule numbers with low computational complexity. Besides, the algorithm works on RGB channels independently, which allows parallelism and is very useful for real-time encryption applications.

Diffusion and confusion are multi-layered processes that hide the spatial relationship of an image, achieved by decimal-level and symbolic-level transformations, respectively. This can also be represented from the uniform histogram and low correlation between adjacent pixels in the encrypted image. These features point to the robustness of the algorithm for the maintenance of image confidentiality with the guarantee of efficient execution.

## Security analysis

The basic requirements of the field of image cryptography are to provide high security and be resistant to various common attacks. These may be different types of cryptanalytic attacks, such as a chosen plaintext/ciphertext attack, statistical attack, differential attack, brute force attack, and others, that have to be faced by a secure design of encryption algorithms. Thus, any encryption technique must try to resist all such threats while being computationally efficient.

### Key space analysis

Key space analysis is concerned with the total number of unique keys available within the encryption scheme to make it resistant against any brute-force attack. In the proposed algorithm, keys have been generated from different components, namely the CDS, KSGP, and certain values for the decimal diffusion and conversion stages. The total key space can be computed based on the number of keys in each category and their precision.

The proposed encryption scheme will use 56 keys divided into the four main components that include the chaotic dynamical system (CDS), the key stream generation procedure, decimal diffusion, and decimal conversion. Here, the CDS with initial conditions and parameters may be represented as $x_0, y_0, z_0, w_0, c_1, c_2, c_3, c_4$ comprises 8 keys. KSGP adds 42 keys, each iteration requires 6 parameters $\kappa _i, \lambda _i, \mu _i, \nu _i, \varrho _i, S_i(-1) \quad \text {for} \quad i \in \{1, 2, \dots , 7\}.$ Decimal diffusion and decimal conversion stages introduce each 3 keys obtained from initial and final pixel values of red, green, and blue channels $RC_{1}(-1), GC_{1}(-1), BC_{1}(-1) \quad \text {and} \quad RC_{5}(-1), GC_{5}(-1), BC_{5}(-1),$ respectively. Each key is represented as a double-precision floating-point number, giving approximately $10^{15}$ possible values per key.

For each category, the total key space is computed as $10^{15}$ raised to the number of keys in that category. Considering CDS, with 8 keys, the key space is $10^{120}$. KSGP, with 42 keys, contributes $10^{630}$. Decimal diffusion and decimal conversion, each with 3 keys, contribute $10^{48}$ each. Merging these together, the total key space is given by $10^{120} \times 10^{630} \times 10^{45} \times 10^{45} = 10^{840}$, see Table 2. This enormous key space far exceeds that required by modern cryptographic standards and provides the algorithm with resistance against brute-force attack.

**Table 2:** Key space.

**Category**	**Keys**	**Per Key**	**Total Key Space**
CDS	$x_0, y_0, z_0, w_0, c_1, c_2, c_3, c_4$ (8 keys)	$10^{15}$	$(10^{15})^8 = 10^{120}$
KSGP	$\kappa _i, \lambda _i, \mu _i, \nu _i, \varrho _i, S_i(-1) ~ \text {for} ~ i \in \{1, 2, \dots , 7\}$ (42 keys)	$10^{15}$	$(10^{15})^{42} = 10^{630}$
Decimal diffusion	$RC_{(1)}(-1), GC_{(1)}(-1), BC_{(1)}(-1)$ (3 keys)	$10^{15}$	$(10^{15})^3 = 10^{45}$
Decimal conversion	$RC_{(5)}(-1), GC_{(5)}(-1), BC_{(5)}(-1)$ (3 keys)	$10^{15}$	$(10^{15})^3 = 10^{45}$
Total key space	$\mathbf {10^{840}}$		

Table 3 compares key space of the proposed method with some other available algorithms. It is clear and obvious that the key space of proposed image encryption algorithm is much superior to others.

**Table 3:** Key space comparison.

Algorithm	Key space
Proposed	$10^{840}$
Norouzi et al.^7^	$10^{56}$
Man et al.^12^	$10^{188}$
Yahi et al.^60^	$10^{84}$
Niu et al.^59^	$10^{70}$
Chen and Ye^61^	$10^{79}$
Benaissi^62^	$10^{704}$

### Sensitivity to secret key

Key sensitivity is one of the important properties of all encrypting algorithms, which essentially means that even a little change in the key will vastly alter the encrypted output. This property makes attacks impossible, wherein an attacker would take advantage of slight key variations to get clues for cryptographic analysis. In order to test the key sensitivity of the presented image encryption algorithm, a perturbation of $10^{-14}$ was added to only one key parameter, namely $y_0$, while keeping the other key parameters $x_0, ~z_0,~ w_0$ unchanged. The perturbed key can be written as $y_0'= y_0 + 10^{-14}.$ Original and perturbed keys were used for encrypting eight standard test images *Lena*, *Baboon, Peppers, Tree, House, Beans, F16*, and *Girl.* The two out of two encrypted images resulting from each test image were then compared by using well-known metrics, such as a Number of Pixels Change Rate (NPCR) and Unified Average Changing Intensity(UACI).

**Table 4:** NPCR and UACI values for key sensitivity analysis.

Images	NPCR (%)			UACI (%)		
	Red	Green	Blue	Red	Green	Blue
Lena	99.6582	99.6368	99.5926	33.3672	33.3832	33.3231
Baboon	99.6140	99.6143	99.6033	33.4353	33.4385	33.4563
Peppers	99.6246	99.6262	99.5758	33.3771	33.4461	33.4459
Tree	99.6338	99.5911	99.5972	33.4657	33.4526	33.4555
House	99.6277	99.5544	99.6368	33.3137	33.2396	33.6816
Beans	99.5819	99.6048	99.5987	33.5689	33.4211	33.5620
F16	99.6094	99.6033	99.6174	33.4296	33.4053	33.4287
Girl	99.6078	99.6048	99.5941	33.3720	33.4696	33.5072
**Average**	**99.6197**	**99.6045**	**99.6020**	**33.4162**	**33.4070**	**33.4826**

Table 4 illustrates the experimental results, which intuitively reflect that the proposed encryption scheme is very sensitive to the key variation. NPCR reflects the percentage of pixel changes between the two encrypted images, and UACI characterizes the mean value of the intensity difference between the two encrypted images. The average values of NPCR for R, G, and B components are 99.6197%, 99.6045%, and 99.6020%, respectively. These results depict that almost all pixel values differed in encrypted images while bringing slight perturbation in the key. Likewise, the average calculated UACI values assessed for red, green and blue channels are 33.4162%, 33.4070% and 33.4826% indicating high level of Pixel intensity differences.

For instance, for *Lena*, the NPCR values are 99.6582%, 99.6368%, and 99.5926% for the red, green, and blue channels, respectively, while the UACI values are 33.3672%, 33.3832%, and 33.3231%. It can also be said about other images, such as Baboon and Peppers, that maybe the proposed algorithm works well for different picture contents. The results authenticate that even the tiniest variation of a single key parameter, lets the encrypted output be highly unpredictable and uncorrelated. Very high values of NPCR and UACI give testimony to the achieved excellent properties in diffusion and sensitivity that are significant for any effective image encryption technique. This justifies the potential of the proposed algorithm toward full resistance against any differential attacks and other analyses in cryptology.

### Avalanche effect

The avalanche effect is a fundamental property of any good cryptographic system: after a slight modification of the plaintext (for example, one bit in the image) or the secret key parameters, a significant change in the ciphertext produced is obtained with unpredictable properties. In other words, changing one bit in either the key or the original image should turn out an entirely different encrypted output. This will ensure that no adversary can deduce any useful structural or statistical information by observing how small variations propagate through the encryption process.

The avalanche effect is an important guarantee against attacks based on the correlation between neighboring image pixels or repeated patterns in image encryption with the proposed 4D hyperchaotic system and Langton’s Ant scrambling. Chaotic systems are sensitive to initial conditions; hence, even minuscule changes in chaotic parameters (such as $x_{0}, y_{0}, z_{0}, w_{0}$) result in widely diverging trajectories and a strong avalanche effect.

Table 5 shows the encryption for eight standard test images, *Lena, Baboon, Peppers, Tree, House, Beans, F16* and *Girl* by changing each parameter of chaotic systems by highly infinitesimal changes of $10^{-13}$ or even $10^{-14}$ order. Columns from the measure of difference-mostly quantified through metrics like the Number of Pixels Change Rate (NPCR) or a similar indicator-remain around 99-99.6%. In other words, with a tiny variation in one parameter, over 99% of the pixels change in the encrypted image relative to its baseline encryption.

**Table 5:** Secret security keys and their difference rates for various images.

Keys	Lena	Baboon	Peppers	Tree	House	Beans	F16	**Girl**
$K_{e_{1}}$ $(x_0' = x_0 + 10^{-13})$	99.6002	99.6030	99.6131	99.6190	99.6350	99.6161	99.6010	99.5771
$K_{e_{2}}$ $(y_0' = y_0 + 10^{-14})$	99.6291	99.6111	99.6092	99.6072	99.6065	99.5951	99.6121	99.6022
$K_{e_{3}}$ $(z_0' = z_0 + 10^{-13})$	99.5842	99.5942	99.5741	99.5881	99.6220	99.6082	99.6081	99.6351
$K_{e_{4}}$ $(w_0' = w_0 + 10^{-14})$	99.5963	99.6081	99.6190	99.6073	99.5964	99.6292	99.6082	99.5833
$K_{e_{5}}$ $(x_0' = x_0 - 10^{-13})$	99.6071	99.6191	99.6191	99.6021	99.5936	99.6124	99.6154	99.5985
$K_{e_{6}}$ $(y_0' = y_0 - 10^{-14})$	99.6072	99.6160	99.5972	99.5962	99.6030	99.6054	99.6167	99.6037
$K_{e_{7}}$ $(z_0' = z_0 - 10^{-13})$	99.6141	99.6110	99.6061	99.6151	99.6041	99.5921	99.6080	99.6139
$K_{e_{8}}$ $(w_0' = w_0 - 10^{-14})$	99.6431	99.5990	99.6218	99.6129	99.6022	99.6114	99.6761	99.6174
**Average**	**99.6102**	** 99.6077**	**99.6075**	**99.6054**	**99.6078**	**99.6099**	**99.6182**	**99.6039**

This gives evidence of truly strong avalanche effect, proving high sensitive dependence on initial condition and unpredictability characters from the 4D hyperchaotic-Lantong’s Ant cryptography approach. Concerning security, this allows one tiny error in assumed key or reconstructed chaotic setup to give a completely different decrypted output image. Thus, this obtained encryption mechanism is resistant toward differential cryptanalysis and full-key space brute-force Key Search attacks as there must be no direct relationship between small variations, which are related to both key/initial stage versus final encrypted images.

### Entropy analysis

Information entropy is a concept of information theory, introduced by Claude Shannon, characterizing the uncertainty or randomness associated with a set of data. In the context of image encryption, entropy quantifies the level of unpredictability in the pixel intensity values of an image. A higher entropy means the distribution of the pixel values will be uniform and random, which in turn makes the identification of patterns or correlations to compromise the encryption scheme harder for the attacker. Entropy in the case of an ideal encryption algorithm for an 8-bit grayscale or RGB image should be near to theoretical maximum of 8-that is, the encrypted image must look like random noise, and hence, will resist any type of statistical and cryptographic attack.

In the presented work, a deep analysis of the entropy for the proposed algorithm of image encryption is performed; the methodology is applied to some standard test images such as *Lena, Baboon, Peppers, Tree, House, Beans, F16,* and *Girl*. Later, the value of entropy for both plain images and encrypted images in RGB is computed by using the following formula for every channel

8 $$\begin{aligned} H(m) = \sum _{i=0}^{2^n-1} p(m_i) \log \frac{1}{p(m_i)}, \end{aligned}$$

where $H(m)$ is the entropy of the message $m$, $p(m_i)$ is the probability of occurrence of the $i^{th}$ symbol, and $n$ is the bit depth of the pixel values. For an ideal encryption scheme, the entropy of the encrypted image should approach the theoretical maximum value, which for an 8-bit image is $H(m) = 8$.

**Table 6:** Comparison of original and encrypted values for various images.

Images	Original			Encrypted		
	Red	Green	Blue	Red	Green	Blue
Lena	7.2507	7.5931	6.9659	7.9974	7.9971	7.9971
Baboon	7.7067	7.4744	7.7522	7.9993	7.9992	7.9994
Peppers	7.3402	7.4770	7.0569	7.9975	7.9972	7.997
Tree	7.2104	7.4136	6.9207	7.9973	7.9973	7.9974
House	6.4311	6.5389	6.2320	7.9969	7.9971	7.9972
Beans	5.7920	6.2195	6.7986	7.9971	7.9968	7.9971
F16	6.7178	6.7991	6.2138	7.9992	7.9992	7.9993
Girl	5.7150	5.3738	5.7117	7.9974	7.9974	7.9973
**Average**	**6.7705**	**6.8612**	**6.7065**	**7.9978**	** 7.9976**	**7.9977**

The entropy values for the encrypted images were much higher than that of the original images, according to the analysis see Table 6. For instance, in *Lena*, the entropy values in the red, green, and blue channels of the original are 7.2507, 7.5931, and 6.9659, respectively. These increase to 7.9974, 7.9971, and 7.9971, respectively after encryption, which is fairly close to the ideal of 8. All test images showed similar trends, their average entropy values being 7.9978, 7.9976, and 7.9977 for the encrypted images in the red, green, and blue channels, respectively.

**Table 7:** Entropy comparison.

Image	Algorithm	Entropy
Lena	Proposed	7.9977
Lena	Norouzi et al.^7^	7.9976
Lena	Malik et al.^11^	7.9971
Lena	Jnana Ramakrishna et al.^23^	7.9987
Lena	Hu et al.^27^	7.9975
Lena	Norouzi and Mirzakuchaki^29^	7.9980
Lena	Benaissi^62^	7.9977

It gives evidence that the values of entropy for the proposed algorithm are near-ideal and prove the effectiveness of it in producing highly random, thus secure, outputs. Thereafter, the encryption scheme introduces indistinguishability of the encrypted images with the random noise by significantly enhancing the unpredictability of the pixel values, hence making them robust against the various attacks of statistical analysis. It infuses high entropy into this algorithm to be reliably used in many secure image encryption applications.

Table 7 depicts the entropy values of various existing methodologies along with the proposed one. The entropy of the proposed algorithm is 7.9977, which is closer to Norouzi et al.^7^ and Benaissi^62^, whereas slightly lesser in comparison to Jnana Ramakrishna et al.^23^ having 7.9987. A very high entropy value depicts the high randomization of the pixel intensities and hence more resistance to statistical attacks.

### Correlation analysis

Correlation reflects the linear dependence of neighboring pixels in an image and is a very important measure concerning how well an encryption algorithm masks the underlying structures. Normally, high values of the correlation between original image adjacent pixels are close to 1, indicating the intrinsic redundancy and smooth regions that may be present in common images. An effective cipher should strongly reduce this correlation, at least toward zero, for all spatial directions: horizontal, vertical, and diagonal.

**Table 8:** Image direction analysis with R, G, B correlations for original and encrypted states.

Image	Direction	R_Original	R_Encrypted	G_Original	G_Encrypted	B_Original	B_Encrypted
Lena	Horizontal	0.94476	0.0014171	0.92038	− 0.0066175	0.87456	0.0059247
Lena	Vertical	0.97035	− 0.00085449	0.95528	0.0015789	0.91262	− 0.012821
Lena	Diagonal	0.92183	0.0010623	0.8994	0.0033688	0.85475	0.0005342
Baboon	Horizontal	0.92306	− 0.0017275	0.86548	− 0.0066308	0.90734	0.00096863
Baboon	vertical	0.86596	− 0.0072012	0.76501	0.0032147	0.88089	0.0036362
Baboon	Diagonal	0.85434	0.0028782	0.73479	− 0.0069473	0.83985	0.00078567
Peppers	Horizontal	0.94221	− 0.0073098	0.95641	− 0.0044152	0.93428	0.0046159
Peppers	Vertical	0.94650	0.0014081	0.95961	0.0047943	0.93806	− 0.0079889
Peppers	Diagonal	0.90879	− 0.0030486	0.92545	0.00010091	0.8959	− 0.0075955
Tree	Horizontal	0.95898	− 0.0082545	0.96868	0.003328	0.96122	0.0094032
Tree	Vertical	0.93606	0.0032045	0.94573	− 0.004682	0.94055	0.00022074
Tree	Diagonal	0.91589	− 0.0019451	0.93175	− 0.0001177	0.92649	0.0079244
House	Horizontal	0.96707	− 0.00071078	0.98052	− 0.00036168	0.98197	0.0045774
House	Vertical	0.93524	− 0.0066258	0.94735	0.0083242	0.97493	− 0.004348
House	Diagonal	0.91263	0.027834	0.93196	0.00084012	0.96251	− 0.0020422
Beans	Horizontal	0.97341	0.000020	0.97075	0.020792	0.97784	− 0.0013507
Beans	Vertical	0.97404	− 0.007921	0.97406	− 0.015595	0.97931	0.0021208
Beans	Diagonal	0.94775	0.010666	0.94698	− 0.020829	0.95827	0.00073674
F16	Horizontal	0.97263	0.0030749	0.95778	0.00019171	0.96397	0.025358
F16	Vertical	0.95681	− 0.0065809	0.96775	− 0.0059401	0.93532	0.012068
F16	Diagonal	0.93433	0.0048622	0.93259	0.0061373	0.91458	0.019773
Girl	Horizontal	0.97788	0.00401	0.9748	− 0.0095197	0.97261	0.012998
Girl	Vertical	0.92938	0.013113	0.91057	0.00036542	0.91297	0.012187
Girl	Diagonal	0.91294	0.0091678	0.89408	0.0037052	0.89579	0.030707

Table 8 presents the correlation coefficients of adjacent pixels in the original versus encrypted images for the R, G, and B channels of *Lena, Baboon, Peppers, Tree, House, Beans, F16*, and *Girl* test images. For each color channel, a separate line is drawn for every direction (horizontal, vertical, diagonal), showing the performance of the cipher in correlating the pixels.

**Original Images:** The correlation coefficients are in the range of approximately 0.73 to 0.98, showing a strong linear dependence of adjacent pixels in all three color channels. Indeed, this is expected in natural images where neighboring pixels are similar in intensity and color.

**Figure 6:**
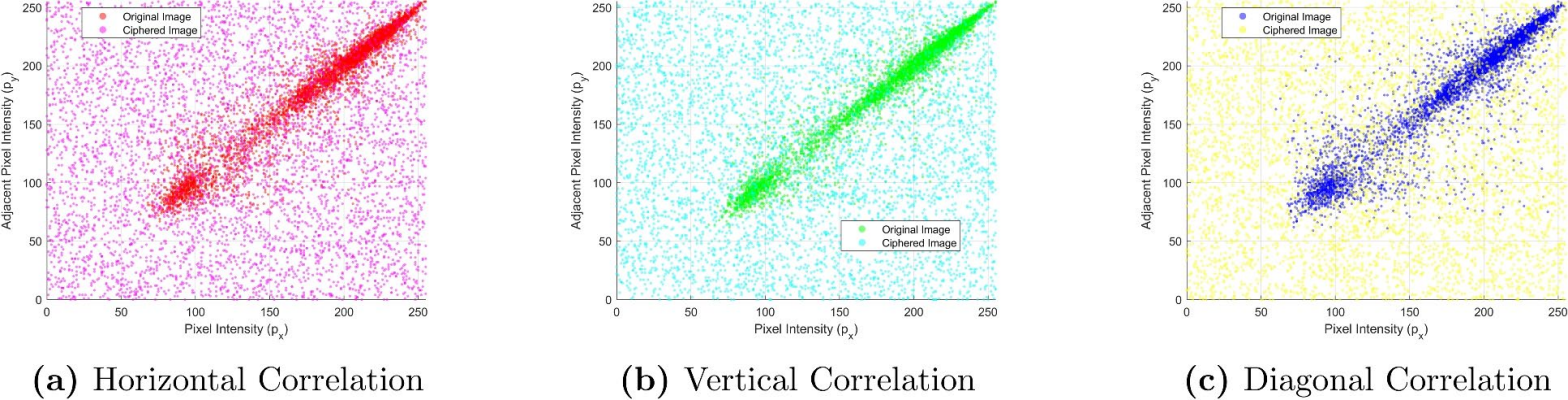
Correlation distribution of adjacent pixels in the original and encrypted images. (**a**) Horizontal, (**b**) Vertical, (**c**) Diagonal.

**Encrypted Images:** This correlation value drops sharply toward zero after encryption in a range of -0.02 to approximately +0.03, there exists no relationship apparently linear in adjacent pixels in different directions (horizontal and vertical and even diagonal direction) or between different color planes R, G, and B. From the near zero values for many directions and multiple color channels, this in fact suggests that the Diffusion-confusion properties that are implemented in the encryption scheme have a very substantial strength.

The scatter plots show the correlation of adjacent pixels in original and encrypted images along horizontal, vertical, and diagonal directions in Fig. 6. In the original image, the intensities of the pixels are highly correlated, as noticed by the tendency of the points to cluster along a line in all three directions. This is because natural images always have highly correlated pixel intensities, since there is always some form of redundancy in the magnitude of the pixels. While the encrypted image has a completely random distribution of pixel intensities, proving that the proposed algorithm disrupts the relationships between pixels effectively. It is observed that the distribution is uniform in horizontal, vertical, and diagonal directions without any trace of patterns. These confirm the strong diffused capability of the algorithm, which wipes out statistical dependencies and resists cryptanalytic attacks like statistical and differential analysis.

**Table 9:** Comparison of correlation coefficients.

Image	Algorithm	Horizontal	Vertical	Diagonal
Lena	Proposed	− 0.00075224	− 0.0018227	0.00108
Lena	Bashir et al.^10^	− 0.00932	0.010248	− 0.005223
Lena	Malik et al.^11^	0.0012	0.0007	− 0.0003
Lena	Jnana Ramakrishna et al.^23^	− 0.00024	− 0.00130	− 0.00013
Lena	Norouzi et al.^29^	− 0.00049	− 0.00057	0.00021
Lena	Su et al.^58^	0.0050	− 0.0001	0.0006
Lena	Bashir et al.^55^	0.023815	− 0.0182048	0.0073787

From Table 9, it is observed that the proposed algorithm achieves the correlation coefficient close to zero in all horizontal, vertical, and diagonal directions more effectively or comparably with other methods. It means that low correlation ensures that the adjacent pixels are strongly decorrelated, which gives rise to a robust security by breaking the spatial relationships. The decorrelation of adjacent pixel values, done by the encryption algorithm itself, is intrinsic in providing resistance against attacks based on statistical patterns. Even in highly correlated image *Lena*, the output cipher texts from the encryption stage do not show meaningful statistical dependencies in any direction in adjacent pixels. Therefore, the 4D hyperchaotic-Langton’s Ant method proposed herein provides an excellent resistance against common attacks that rely on exploiting the local or global structure of the image.

### Histogram

A histogram is a graph plotting the distribution of pixel intensities in an image. Within the context of encryption, the histogram is an important tool to visually and quantitatively determine whether or not the algorithm is sufficiently masking the spatial redundancies and frequency patterns present in the original image. Ideally, an unencrypted image should have peaks and valleys in its histogram corresponding to dominant colors or intensities. For instance, a bright image will have more pixel counts concentrated at higher intensity values, while darker images skew toward lower intensities. However, for the robustness of security, a histogram of an encrypted image should appear as uniform as possible, no single intensity or range of intensities should dominate, hence giving evidence that the cipher has successfully diffused and confused the original pixel values.

**Figure 7:**
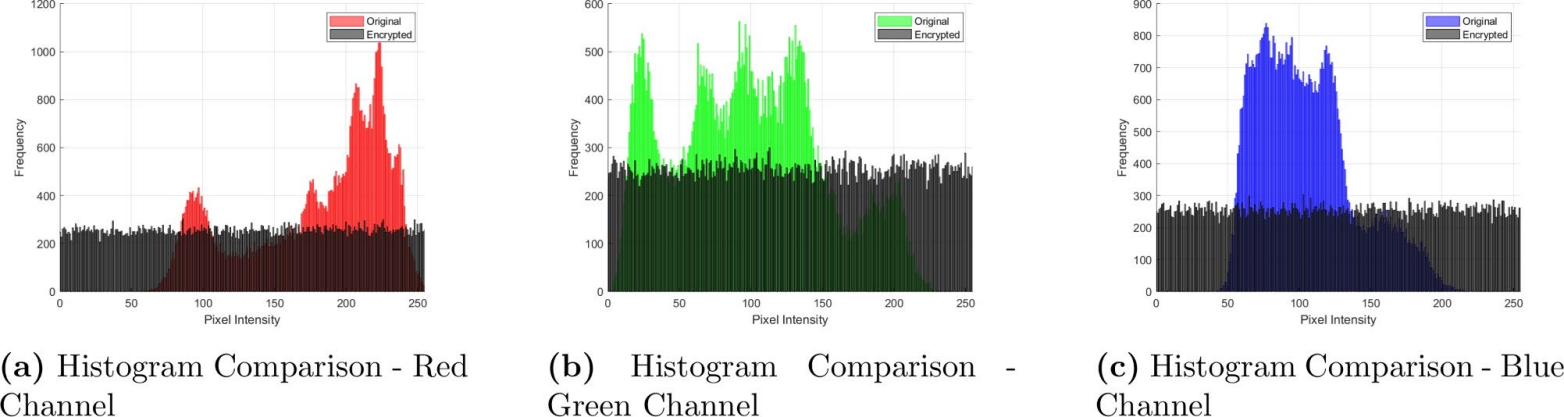
Histogram comparison of red, green, and blue channels for original and encrypted lena images.

The histograms of the *Lena* image before and after encryption are given in Fig. 7. The histograms of the red, green, and blue channels (for instance, Fig. 7a) of the original image have a number of evident peaks, which means the intensity of pixels is not uniformly distributed. These kinds of patterns in the original image may provide statistical clues for attackers. On the other hand, it can be observed from Figs. 7a–c that the histograms of the encrypted image are uniformly distributed. The uniformity of distribution of pixel intensities shows that the encryption algorithm diffuses them effectively enough to mask any inherent structure and features that the image may possess. Hence, it will be highly impossible for an intruder to identify from this either the content of the image or the encryption key.

An important statistical measure with respect to the spread of these histogram values is the *variance*. Variance essentially a measure of how much the data points (pixel count) deviate from their mean value. In the following Table 10, variance has been calculated for nine different keys $K_{e_1}$ through $K_{e_8}$ for several standard test images-*Lena, Baboon, Peppers, Tree, House, Beans, F16*, and *Girl*. Larger or smaller variance can then show the dispersion of the image’s pixel distribution over the range of possible intensities. Commonly, a well-encrypted image will have an appropriate value of variance that does not suggest clustering or strong peaks, which again would claim the hypothesis of uniformity in pixel value distribution.

**Table 10:** Key performance data.

Image	${K_{e_{_1}}}$	${K_{e_{_2}} }$	${K_{e_{_3}}}$	${ K_{e_{_4}} }$	${K_{e_{_5}}}$	${K_{e_{_6}}}$	${ K_{e_{_7}}}$	${ K_{e_{_8}}}$	${K_{e_{_9}} }$
Lena	5473	5461.2	5455.8	5454.7	5455.7	5455.5	5450.1	5453.7	5468.7
Baboon	5463.7	5465.2	5466.9	5474.7	5465.8	5461	5456.2	5460.1	5454.8
Peppers	5454.3	5444.7	5467.8	5456.7	5461.6	5466.5	5471.2	5460.7	5474.9
Tree	5453.9	5466.9	5454.4	5458	5459.3	5465.8	5466.6	5455.7	5459.7
House	5444.4	5458.9	5459.8	5454.3	5464.1	5474	5470.9	5460.7	5469.1
Beans	5487	5455.6	5469.7	5457.3	5477.3	5461.4	5462.6	5458.5	5435.4
F16	5456.5	5459.4	5462.9	5463.7	5465.9	5457.3	5464.9	5466.2	5463.6
Girl	5470.8	5459.9	5449	5461.6	5482.2	5466.9	5456	5451.8	5441.2
Average	5462.9	5459	5460.8	5460.1	5466.5	5463.5	5462.3	5458.4	5458.4

Through these variance results and visual histogram inspections, it becomes clear that the proposed encryption approach spreads out pixel intensities effectively, rendering the cipher robust against statistical attacks aimed at uncovering patterns in pixel distributions. Consequently, a near-uniform histogram signals a higher level of security, since each encrypted pixel is indistinguishable in terms of intensity likelihood-a crucial hallmark for modern image encryption schemes.

### Chi-Square analysis

Chi-square analysis is a powerful statistical tool used in order to test the likelihood that an image’s pixel intensities come from some common (usually uniform) distribution. More specifically, we model the occurrence frequency of each pixel intensity level, from 0 to 255, for both the *original* and *encrypted* images, and compare the resulting sets of observed frequencies against the theoretical uniform distribution. The chi-square value, $\chi ^2$, is the sum of the squared deviations of observed counts from expected counts, normalized by the expected count of each intensity. Formally, for every intensity level $i$, if $P_i$ is the observed count and $C_i$ is the expected count, then

9 $$\begin{aligned} \chi ^2 = \sum _{i=0}^{255} \frac{(P_i - C_i)^2}{C_i}. \end{aligned}$$

In general, the lower the chi-square values, the closer the observed distribution is to the theoretical uniform distribution. The higher values mean larger deviation from uniformity.

Table 11 reports the chi-square values in the R, G and B channels of several images before and after encryption. It can be seen that the original images have considerably larger chi-square values. The R-channel of the original *F16* reaches $6.7842\times 10^5$ and the G-channel of *Girl* is $8.6097\times 10^5$. Large value indicates that the pixel intensities of these plain images cluster around certain intensity levels, hence they are far from uniform distribution.

In contrast, the chi-square values of each channel of the encrypted images are considerably lower, often a couple of orders of magnitude smaller. For example, the R-channel of *Lena* drops from $63888$ in the original version to only $235.54$ after encryption. This sharp drop confirms that the ciphered images lose the characteristic peaks of the original histograms and approach a more flattened, uniform-like distribution. This decorrelating and histogram flattening are, in other words, intrinsic to good encryption, as it minimizes clues an attacker might use about the underlying image content.

**Table 11:** Chi-square values for original and encrypted images.

Image	$R_{{{Original}}}$	$R_{{{Encrypted}}}$	$G_{{{Original}}}$	$G_{{{Encrypted}}}$	$B_{{{Original}}}$	$B_{{{Encrypted}}}$
Lena	63888	235.54	28546	274.14	86488	269.83
Baboon	82840	257.66	142810	280.60	79943	231.94
Peppers	53024	229.96	88272	258.08	125180	268.40
Tree	81371	242.08	57009	241.49	129820	236.23
House	258580	282.01	299160	260.36	394040	256.30
Beans	537500	275.40	349250	295.27	129760	266.78
F16	678420	276.71	682500	307.26	1107900	260.93
Girl	790830	233.09	860970	237.46	620380	248.04

Chi-square analysis thereby presents quantitative evidence that indeed, the encryption scheme is effectively redistributing the pixel intensities to be much more consistent with a uniform distribution. High chi-square values in the original images only validate their naturally uneven intensity structure, while the vastly reduced chi-square values in these encrypted images show successful mask, diffusion, and confusion properties. From a security power standpoint, a near uniform histogram means each pixel intensity is Equi-probable, and hence any pattern that was there is now concealed, thus not being exploited.

### Differential cryptanalysis

The main principle of differential cryptanalysis is checking the sensitivity of an encryption algorithm to small perturbations in the input data. In the image encryption framework, this method investigates how a minute modification in the plain image-for example, the flipping of one pixel-affects the resulting encrypted image. A robust cipher is expected to diffuse such a tiny change to the whole ciphertext, so that the encrypted result is very different from the version generated by the unmodified plaintext. Two commonly used metrics to quantify this behavior are the Number of Pixels Change Rate (NPCR) and the Unified Average Changing Intensity (UACI).

Let us take two encrypted images obtained using nearly identical inputs, $E_{1}$ and $E_{2}$. Let one be obtained from the unaltered image and the other from the very same image with a single pixel flipped. Let $\textrm{rows} \times \textrm{cols}$ be the image dimension. To get NPCR, define an indicator function $\Delta (i, j)$ which is $1$ provided the two encrypted pixels are different at coordinates $(i, j)$ or else $0$. Then, NPCR will be

10 $$\begin{aligned} \mathcal {N}(E_{1}, E_{2}) = \frac{\sum _{i,j} \Delta (i,j)}{\textrm{rows} \times \textrm{cols}} \times 100\%, \end{aligned}$$

where the larger the NPCR is, it denotes that more pixel positions have been changed, which means the effect of diffusion is stronger. As a matter of fact, all NPCR values larger than 99% can already be considered an evidence of robustness in the process of encryption.

UACI, on the other hand, measures how much the pixel intensities differ, on average, between $E_{1}$ and $E_{2}$. Let $\wp$ be the maximum possible pixel intensity difference (commonly 255 for 8-bit images). UACI is defined by

11 $$\begin{aligned} \mathcal {U}(E_{1}, E_{2}) = \frac{\sum _{i,j} \left| E_{1}(i,j) - E_{2}(i,j) \right| }{\wp \times \textrm{rows} \times \textrm{cols}} \times 100\%. \end{aligned}$$

This metric reflects how large the absolute differences between corresponding pixel intensities become. Higher UACI values indicate that even when a single pixel is changed in the plain image, it causes widespread intensity alterations in the cipher image.

**Table 12:** Differential cryptanalysis results.

Image	NPCR	UACI
Lena	99.581	33.511
Baboon	99.613	33.503
Peppers	99.467	33.485
Tree	99.631	33.521
House	99.586	33.431
Beans	99.577	33.542
F16	99.583	33.426
Girl	99.876	33.567

Table 12 shows the NPCR and UACI values of several standard test images in the differential cryptanalysis attack. Most of the images’ NPCR is close to 99.5% while the UACI value is close to 33.5%, confirming most strong encryption algorithms have high sensitivity to small changes of the original image. What’s more, *Girl * can reach an NPCR value as high as $99.876\%$, and its UACI value is $33.567\%$, proving that the encryption algorithm can be very sensitive to small changes. In general, the test images confirm that one pixel flipping in the plain image results in large, apparently unpredictable changes in the encrypted version. Consequently, a high NPCR and UACI value means excellent sensitivity of the encryption scheme to make it resistant against attacks that are based on partial knowledge of the plaintext or incremental changes.

In general, differential cryptanalysis gives the important idea about how effectively an encryption algorithm spreads small changes in the input data to the ciphertext. The large NPCR and UACI values ensure that any manipulation or guessing of segments of the plain image will result in a totally different encrypted output, hence strengthening the security of the cipher.

**Table 13:** Comparison of NPCR and UACI values.

Algorithm	NPCR	UACI
Proposed	99.6087	33.4352
Toughi et al.^9^	99.6001	33.480
Bashir et al.^10^	99.6163	33.4630
Malik et al.^11^	99.630	33.5296
Chen et al.^14^	99.62	33.48
Fu et al.^17^	99.61	33.48
Wang et al.^21^	99.611	33.453
Jnana Ramakrishna et al.^23^	99.610	33.480
Hu et al.^27^	99.5991	33.4650

In Table 13, we have compared the NPCR and UACI values of the proposed scheme with various existing algorithms present in the literature. One can observe that the proposed method achieves competitive results, with NPCR as high as 99.61% and UACI of about 33.44%. These numbers demonstrate that even a very small change in the plain text image introduces significant changes to the cipher text image.

### Mean square error (MSE) and peak signal to noise ratio analysis (PSNR)

PSNR is usually adopted for comparing the fidelity between two images, where one usually represents the original image, and the other one is somehow perturbed or altered. For this work, the altered version is the encrypted, or ciphered, image that now represents how much the encryption algorithm deviates from the original regarding the pixel intensity values. PSNR originates from the Mean Squared Error, which is the mean squared difference between the original and the encrypted images. The MSE may be formally defined as

12 $$\begin{aligned} \text {MSE} = \frac{1}{M \times N} \sum _{i,j} \left( P_{0}(i,j) - P_{1}(i,j) \right) ^2, \end{aligned}$$

where $M$ and $N$ are the width and height of the image, respectively. $P_{0}(i,j)$ and $P_{1}(i,j)$ denote the intensity values of the pixel at position $(i, j)$ in the original and ciphered images, respectively. A larger MSE indicates a greater average difference between the two images.

From the MSE, the PSNR is computed as

13 $$\begin{aligned} \text {PSNR} = 20 \log _{10} \left( \frac{255}{\sqrt{\text {MSE}}} \right) \, \text {dB}. \end{aligned}$$

Here, 255 is the maximum possible pixel intensity in an 8-bit grayscale channel. A high value of PSNR usually indicates that the two images are highly similar; but for image encryption, it should be such that the cipher image is quite different from the original one. Therefore, high MSE provides low PSNR, which, in fact, depicts that the encryption algorithm introduces severe distortion compared to the original image. This in return would mean a stronger security, as the cipher will increasingly be hard for an adversary to infer anything meaningful.

Table 14 gives PSNR values (in decibel) of several standard test images - *Lena, Baboon, Peppers, Tree, House, Beans, F16 * after encryption with the proposed technique. PSNR is the ratio between maximum power of an original signal and that of noise or error value added to the signal. Clearly lower PSNR value around 7-10 dB indicates a greater perturbation over the content which means there is little scope of getting back the features from these images without going for an appropriate decryption. The outcome therefore establishes that the given visual content has been reasonably blurred by the proposed encryption technique.

**Table 14:** Comparison of the PSNR values.

Image	Lena	Baboon	Peppers	Tree	House	Beans	F16	Girl
Algorithm	PSNR (dB)							
Proposed	8.6347	8.7763	8.0697	8.1448	8.9166	8.6436	7.9886	9.9500
Malik et al.^11^	7.8694	8.7855	9.0991	8.6982	8.8163	8.4897	8.1482	9.8564
Bashir et al.^11^	7.8616	8.7707	9.1013			8.5221		
Jnana Ramakrishna et al.^23^	7.6548							
Ye et al.^25^			37.5465		37.6543			
Taneja et al.^28^	9.3008							9.5639
Norouzi et al.^29^	8.4925	9.4326	8.8700				7.9684	

### Noise and occlusion attacks

The proposed encryption scheme resists many cryptographic attacks like known-plaintext (KPA), chosen-plaintext (CPA), differential, and occlusion attacks through the use of robust diffusion and confusion. Unpredictable encryption patterns are created by chaotic key generation so that even minor plaintext variations produce vastly different ciphertexts, and it becomes computationally infeasible for the attacker to extract meaningful information without the proper decryption key.

**Figure 8:**
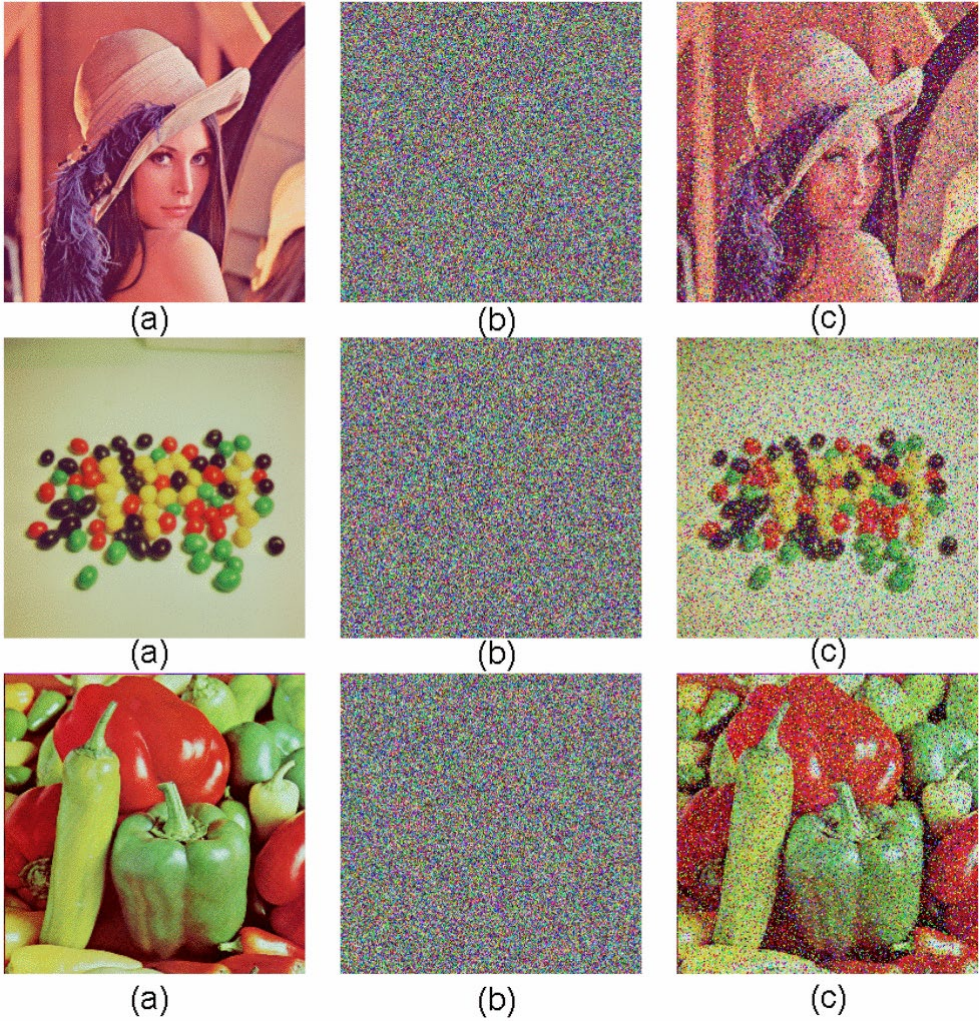
(**a**) Original (**b**) Cipher images with 0.2 density; (**c**) Decrypted images.

For verification, we used the proposed algorithm on standard test images (Lena, Peppers, and Beans) with added salt & pepper noise (density = 0.2) and occlusion attacks prior to encryption. Experimental results demonstrated that the encryption process transforms the originals into highly randomized cipher images with no visible structure, while the decryption process restores the originals with minimal distortion even when the input is intentionally corrupted.

**Figure 9:**
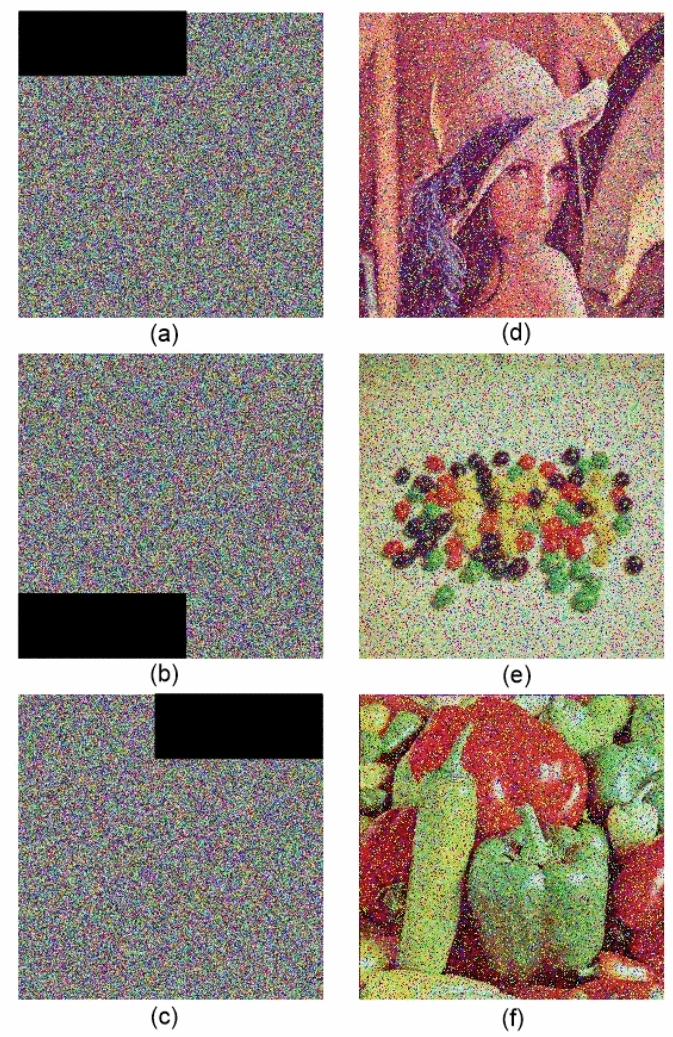
(**a**–**c**) cipher images with data loss. (**d**–**f**) decrypted images.

For the sake of clarity, we display images see Figs. 8 in an ordered sequence: (a) the original images, (b) the cipher images with noise and (c) the decrypted images. Figure 9a–c draw the cipher version of Lena, Beans, and Peppers with data loss attack, and the Fig. 9d,e corresponding decrypted images. This arrangement clearly shows the ability of the algorithm to encrypt images under adverse conditions, maintaining the integrity of the information and thus making it an effective solution for secure image transmission.

### Computational complexity

Computational complexity for the proposed encryption scheme was studied by dividing the process into various stages like image preprocessing, pixel reordering, chaotic keystream generation, symbolic encoding with Langton’s Ant, multi-stage diffusion, and final image reconstruction. For an $m\times n$-sized image, each of these processes is performed for each pixel, resulting in a complexity proportional to *O*(*mn*) for every step. When all the stages are combined together, the overall complexity amounts to about $27 \cdot m \cdot n.$. This linear time complexity indicates that even with the multi-layered structure and utilization of various chaotic transformations, the proposed scheme is computationally efficient and scalable for the handling of big-sized images.

## Conclusion

The proposed image encryption algorithm effectively integrates the unpredictability of a four-dimensional chaotic system with the symbolic manipulation capability of Langton’s Ant cellular automaton, hence providing a very secure and efficient framework for image cryptography. In this regard, the algorithm ensures strong diffusion and confusion properties, considered crucial for resisting statistical and differential cryptographic attacks by integrating multi-level transformations such as decimal diffusion, chaotic permutation, and symbolic encoding. The inherent sensitivity of the chaotic system to initial conditions, along with the dynamic rule-based encoding by Langton’s Ant, ensures that the variations in the ciphertext image are going to be quite secure and immensely varied. The key performance evaluations demonstrate the resistance of the algorithm to various types of cryptanalysis. This scheme has an enormously large key space of $10^{840}$, far larger than the modern cryptographic threshold, hence it can be very resistant against any kind of brute-force attack. Entropy analysis reveals that the pixel intensity distribution of the encrypted images is almost random and reaches a value very close to the theoretical maximum value 8 for 8-bit images, proving the capability of the algorithm to generate indistinguishable ciphertexts. Moreover, the proposed algorithm is effective in eliminating the spatial dependency between adjacent pixels, as confirmed by the correlation analysis showing a correlation near to zero in all horizontal, vertical, and diagonal directions. This means the statistical pattern in the encrypted image is not distinguishable. The scheme, due to the modular nature of the chaotic and symbolic operations, ensures computational efficiency for parallel processing in real-time applications. It also shows a very good avalanche effect: a small change in either plaintext or any of the key parameters results in a drastic change in the ciphertext produced. This property enhances the security of the encryption scheme by ensuring high sensitivity to inputs. The proposed algorithm represents a milestone in chaos-based image encryption, offering strong security with computational practicality. Such an algorithm is sure to meet almost all complex cryptographic requirements and might turn out to be very effective in a number of applications, including secure communications, medical imaging, and military operations involving the transmission of multimedia data. Further research may be directed to the extension of this framework to higher-dimensional chaotic systems and the optimization of its realization under resource-constrained conditions.

Our scheme guarantees high security, multi-stage chaotic keystream generation, symbolic encoding, and multi-stage diffusion incur more computations compared to simpler schemes. This may have implications on real-time applications with high-speed demands. The scheme could be applied to bigger images of size 512$\times$512 and 1024$\times$1024, but the bigger volume of data leads to longer processing times, rendering it inefficient for large-scale multimedia encryption without optimization. The scheme’s security greatly depends on the initial chaotic parameters. While the scheme is highly sensitive to small parameter changes, improper parameter selection may lead to performance degradation with the necessity of careful tuning for different applications. Using the proposed method of encryption on constrained platforms like the IoT and embedded systems may prove to be challenging due to its higher computations, with the need for additional optimization to fit lightweight applications.

## Data Availability

All the data used to finding the results is included in the manuscript.
